# Application of Thermal Response Measurements to Investigate Enhanced Water Adsorption Kinetics in Ball‐Milled C_2_N‐Type Materials

**DOI:** 10.1002/open.202200193

**Published:** 2022-12-13

**Authors:** Shengjun Du, Desirée Leistenschneider, Jing Xiao, Jan Dellith, Erik Troschke, Martin Oschatz

**Affiliations:** ^1^ Institute for Technical Chemistry and Environmental Chemistry Center for Energy and Environmental Chemistry Jena (CEEC Jena) Friedrich-Schiller-University Jena Philosophenweg 7a 07743 Jena Germany; ^2^ School of Chemistry and Chemical Engineering South China University of Technology Guangzhou 510641 China; ^3^ Department Competence Center for Micro- and Nanotechnologies Leibniz Institute of Photonic Technology Albert-Einstein-Straße 9 07745 Jena Germany

**Keywords:** ball milling, C_2_N-type materials, diffusion, particle size, water adsorption

## Abstract

Sorption‐based water capture is an attractive solution to provide potable water in arid regions. Heteroatom‐decorated microporous carbons with hydrophilic character are promising candidates for water adsorption at low humidity, but the strong affinity between the polar carbon pore walls and water molecules can hinder the water transport within the narrow pore system. To reduce the limitations of mass transfer, C_2_N‐type carbon materials obtained from the thermal condensation of a molecular hexaazatriphenylene‐hexacarbonitrile (HAT‐CN) precursor were treated mechanochemically via ball milling. Scanning electron microscopy as well as static light scattering reveal that large pristine C_2_N‐type particles were split up to a smaller size after ball milling, thus increasing the pore accessibility which consequently leads to faster occupation of the water vapor adsorption sites. The major aim of this work is to demonstrate the applicability of thermal response measurements to track these enhanced kinetics of water adsorption. The adsorption rate constant of a C_2_N material condensed at 700 °C remarkably increased from 0.026 s^−1^ to 0.036 s^−1^ upon ball milling, while maintaining remarkably high water vapor capacity. This work confirms the advantages of small particle sizes in ultramicroporous materials on their vapor adsorption kinetics. It is demonstrated that thermal response measurements are a valuable and time‐saving method to investigate water adsorption kinetics, capacities, and cycling stability.

## Graphical abstract

**Thermal response measurements** as well as volumetric water physisorption measurements are applied to study the influence of particle sizes on water adsorption capacity and kinetics. For this, a controlled thermal condensation process followed by a mechanical treatment using a ball mill is applied to synthesize C_2_N‐type carbon materials with smaller and larger particle sizes. The nitrogen‐rich surfaces and narrow distributed micropores endow them with remarkable hydrophilic features. Using the InfraSORP technique, a fast and accurate assessment of these properties is demonstrated.

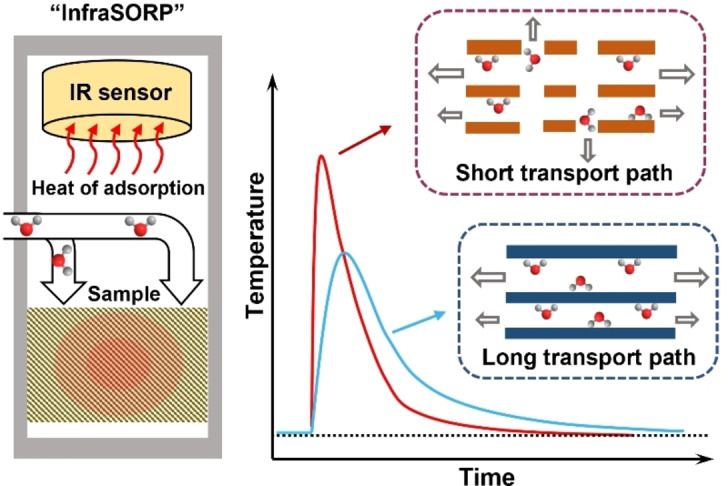

## Introduction

Global water shortage is one of the most imperative but challenging issues in the 21^st^ century due to a steadily increasing demand for clean water.^[1]^ Water harvesting by porous solids is a promising way to provide freshwater in water‐stressed regions and/or remote areas.^[2]^ Among currently known porous materials for water adsorption, silica gels, zeolites, and some representatives of metal–organic frameworks (MOFs) with inorganic building units exhibit high moisture capture capacity over a wide range of humidity values due to the formation of strong hydrogen bonds and dipol‐dipol interactions with water molecules.^[3]^ However, the effectiveness of such established “water capture” sorbents is often limited by their poor stability, limited water adsorption capacity/kinetics, energy‐intensive regeneration, broad pressure range for water uptake, high cost, or the need for non‐abundant/toxic precursor materials or molecules for their synthesis.

Carbon‐based adsorbents naturally provide outstanding durability and tunable porosity. Pristine carbons can be decorated with heteroatoms to enhance their polarizing properties and to introduce specific adsorption sites.^[4]^ In consequence, they have been widely exploited for gas adsorption.^[5]^ Traditional porous carbonaceous materials such as activated carbon, carbon nanotubes, or carbon molecular sieves have rather hydrophobic surfaces due to the inherently low affinity between unpolar sp^2^‐hybridized carbon and water molecules.^[6]^ On such surfaces, adsorption processes are dominated by low‐enthalpy van‐der‐Waals (dispersion) forces and water‐water interactions remain dominating. Thus, the capture of traces of water at low humidity is not possible with such hydrophobic carbons.

In recent years, considerable research efforts have demonstrated that the incorporation of heteroatoms into nanoporous carbon materials can lead to a hydrophilic surface due to the possibility of hydrogen‐bond formation or the formation of donor‐acceptor couples with water molecules.^[7]^ The most widely investigated heteroatom is nitrogen, but other elements with an electronegativity that differs from carbon and/or Lewis‐acidic/Lewis‐basic character (e. g., oxygen, sulfur, phosphorus, or boron) have also been investigated.^[8]^ Unfortunately, the required nitrogen functionalities with electron‐donating character are usually not sufficiently stable during carbonization at high temperature. It is therefore difficult to obtain carbon materials decorated with a high density of heteroatoms in defined binding motives by applying post‐synthetic precursor routes or simple heteroatom‐containing carbon precursors such as glucosamine which do not follow a well‐defined condensation mechanism.^[9]^ Controlled condensation of precursor molecules that already contain the heteroatoms in the desired binding configuration appears to be a more suitable method to obtain nitrogen‐rich carbon compounds with defined chemical architectures.^[10]^ Recently, a material with a near‐perfect C_2_N‐type composition and a high content of pyrazinic nitrogen obtained by the one‐step thermal condensation of a molecular hexaazatriphenylene‐hexacarbonitrile (HAT‐CN) precursor has been reported.^[11]^ The obtained stable C_2_N structure consists of particles with tens of micrometers in size and further contains small micropores with a narrow size distribution, thus combining high surface area with ultra‐hydrophilic surface properties.[^7a^, ^12^] However, the strong affinity to water and the small micropores apparently lead to slow intraparticle diffusion rates.

The diffusion process of water molecules in nanoporous carbon particles mainly comprises four steps:^[13]^ adsorption to the outer surface of sorbent particles (i), diffusion into internal nanopores (ii), interaction with pore walls and their binding sites (iii), and finally release to external‐pore environment (iv). As a consequence, narrow micropores and high‐energy adsorption sites are favorable for the confinement of water, but they can restrict the transport of water molecules through the adsorbent particles at the same time. One way to achieve enhanced kinetics in gas adsorption processes is the reduction of particle size. This shortens the transport paths and further introduces new channels into carbon. Among the available methods for fragmentation, mechanical force provided by ball milling is an attractive “top‐down” way to reduce particle sizes with moderate energy input.^[14]^

In order to apply this principle to carbon‐based water adsorbents, two materials decorated with pyrazinic nitrogen were prepared by thermal condensation of HAT‐CN at 550 and 700 °C. A ball milling treatment was then applied to reduce the particle size of the as‐obtained materials. The morphology and chemical microstructures of C_2_N‐type materials before and after ball milling were investigated. The water adsorption performance was studied by thermal response measurements based on optical calorimetry (designated as “InfraSORP”),^[15]^ and static isotherms, allowing for the accurate assessment and comparison of water kinetics and adsorption capacity. The InfraSORP technology has recently been used to track adsorption processes of polar molecules such as water and ammonia,[^15b^, ^16^] but to our best knowledge, this work represents the first use of the InfraSORP apparatus as a tool to evaluate the water adsorption kinetics within hydrophilic carbon networks. A comprehensive analysis of the obtained findings revealed that the breakage of particles using ball milling is responsible for shortening the length of diffusion paths for water and thus reduces transport limitations compared with pristine C_2_N materials. While the aim of this work is to concentrate on the investigation of the water adsorption kinetics in C‐HAT‐550 and C‐HAT‐700 as model compounds and to show the applicability of thermal response measurements to resolve their differences before and after ball milling. We do not aim to further investigate the particular influence of nitrogen doping on the water adsorption properties of the materials as such which has been part of numerous recent studies.^[17]^

## Results and Discussion

**Figure 1:**
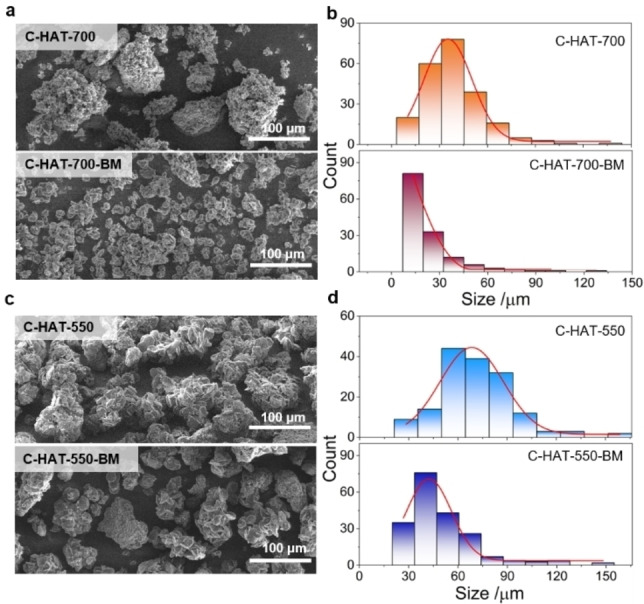
Representative SEM images and the corresponding histograms of measured particle size with a Gaussian fit of (a–b) C‐HAT‐700 before and after ball milling as well as of (c–d) C‐HAT‐550 before and after ball milling.

The micromorphological characteristics of the investigated set of C_2_N‐type materials were studied using scanning electron microscopy (SEM, Figures 1 and S1). The pristine C‐HAT‐700 and C‐HAT‐550 samples without further treatment appear with a discoid‐like shape and seem to be bundled together randomly. The ball milling treatment reduced the proportion of the large particles and resulted in a smaller size due to the fragmentation of bundles and plates. Histograms of characteristic size distribution show that the value of particle sizes decreased from ca. 17–60 μm for C‐HAT‐700 to ca. 7–20 μm for C‐HAT‐700‐BM, and ca. 50–93 μm for C‐HAT‐550 to ca. 20–60 μm for C‐HAT‐550‐BM. The decrease of particle size after ball milling on a bulk scale was further confirmed by static light scattering (SLS) measurements (Figure S2). As expected, a clear decrease of the particle sizes has been observed for both samples after ball milling. It should be mentioned that particle sizes, on average, are larger from the SLS measurements as compared to SEM findings due to the possible formation of aggregates under the conditions of the measurements.

**Figure 2:**
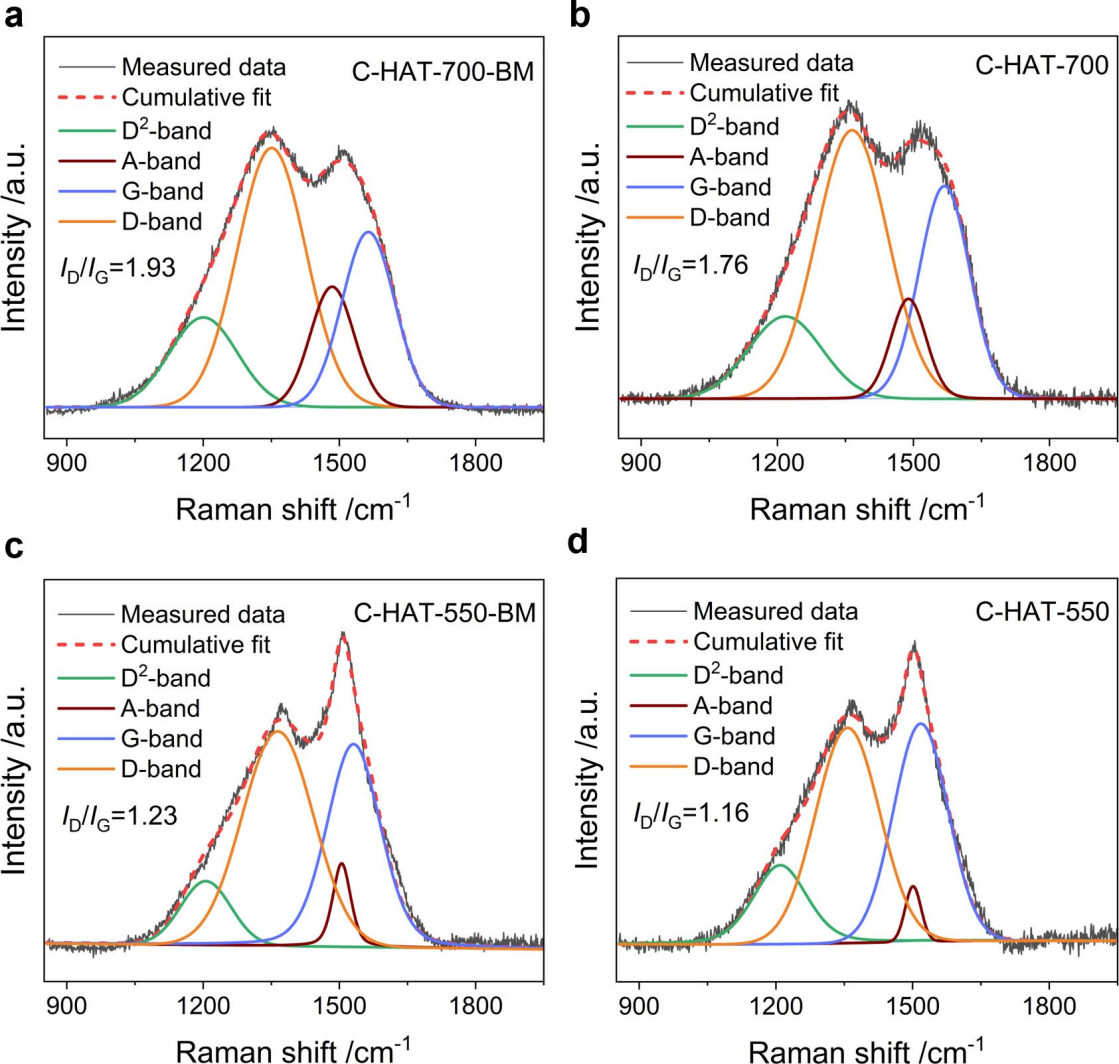
Deconvoluted Raman spectra of (a) C‐HAT‐700‐BM; (b) C‐HAT‐700; (c) C‐HAT‐550‐BM; (d) C‐HAT‐550.

The ordering degree of the carbon structures was characterized by powder X‐ray diffraction (XRD) measurements (Figure S3). No traces of zirconia impurities from the planetary ball mill can be detected. Only one broad reflex located at about 27° can be observed as is typical for highly microporous carbons. This corresponds to the (002) planes of π‐π stacking between adjacent carbon layers, and it indicates the inhomogeneously stacked graphene sheets and a lack of pronounced long‐range ordering in the carbon structure.[^8c^, ^18^] To further estimate the change of defects and structural order before and after ball milling, Raman spectra were recorded and fitted by using a four‐band model, including the D^2^‐, D‐, A‐, and G‐bands (Figure 2).^[19]^ The disordered D‐band centered at around 1330 cm^−1^ is associated with vibrations of sp^2^ carbon atoms in aromatic rings located at the defects and the edge plane. The graphene‐like G‐band at around 1580 cm^−1^ corresponds to vibrations between sp^2^ carbon atoms organized in chains and rings.[^14a^, ^20^] Although the general shape of the spectra remains comparable after ball milling, the intensity ratio (*I*_D_/*I*_G_) visibly increases after ball milling (1.76 to 1.93 for C‐HAT‐700 and 1.16 to 1.27 for C‐HAT‐550), indicating an increased concentration of defects and edges within the carbon structure when smaller particles are present. Therefore, it can be found that the A‐ band, originating from the amorphous carbon fraction, increased in intensity as compared to the D‐ and G‐bands after ball milling for both condensation temperatures.

X‐ray photoelectron spectra (XPS) were recorded to elucidate the different bonding features of nitrogen and carbon atoms in the as‐synthesized samples (Figures S4‐7). Deconvolution of the N1s spectra reveals that no obvious change can be observed in the relative peak areas of corresponding nitrogen functional groups as a result of ball milling. It is worth noting that, through fitting of the acquired C1s spectra, the ratio of the sp^2^ C drops from 35.7 % in C‐HAT‐700 to 21.0 % for C‐HAT‐700‐BM and 18.8 % in C‐HAT‐550 to 15.0 % for C‐HAT‐550‐BM. Such a change suggests a partial destruction of the ordered carbon structure during mechanochemical treatment which is consistent with the Raman results discussed before. The elemental composition of the particle surface obtained from the XPS survey spectra shows that ball milling slightly reduces the C/O atomic ratio of both samples as a result of the binding of oxygen species to the carbon‐nitrogen matrix (Table S1). The nature of the oxygen species on the surface does not change in response to the ball milling procedure (Figure S7). Additionally, the C/N atomic ratio of pristine C‐HAT‐550 is 2.1, suggesting a nearly perfect C_2_N‐type stoichiometry. This value further increased to 2.5 for the C‐HAT‐700 sample with higher condensation temperature due to the lower stability of heteroatoms with increasing temperature and ongoing condensation of HAT‐CN leading to the removal of further nitrile groups. After ball milling, the C/N ratio of C‐HAT‐700 and C‐HAT‐550 did not change significantly and a high nitrogen content remained.

**Figure 3:**
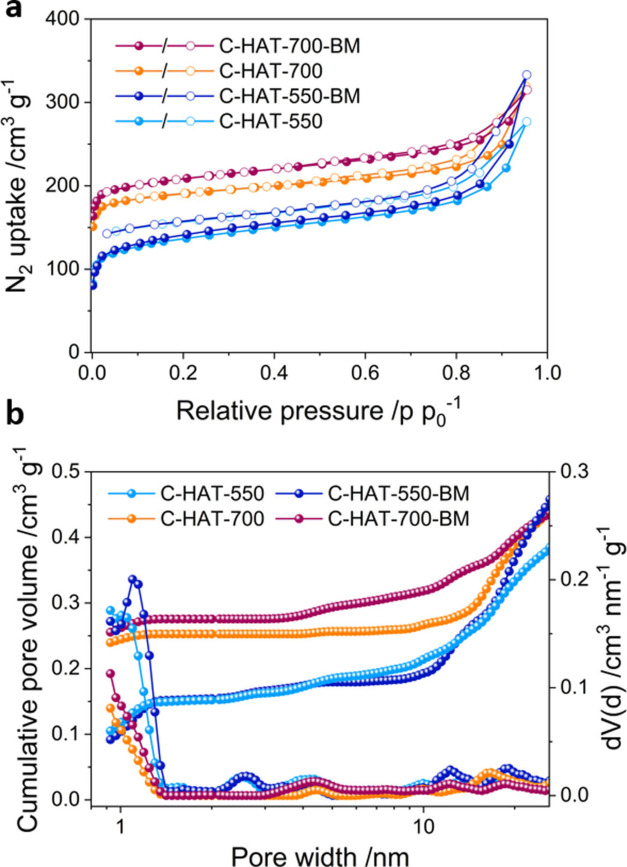
(a) N_2_ adsorption‐desorption isotherms at 77 K for C‐HAT‐700 and C‐HAT‐550 with and without ball milling (Solid and empty circles represent adsorption and desorption data, respectively); (b) Cumulative pore volume and pore size distributions calculated from the QSDFT model.

The pore structure of the materials was investigated using nitrogen physisorption measurements at 77 K (Figure 3a). Both samples exhibit an isotherm with a type I shape according to the IUPAC classification with a steeply increasing uptake in the low‐relative pressure region, which is a typical character of materials with small micropores.^[21]^ At high relative pressure (p p_0_^−1^>0.8), the nitrogen uptakes slightly increase, suggesting the existence of macropores and/or interparticular voids in the materials. After ball milling, C‐HAT‐700 and C‐HAT‐550 showed a slight increase in nitrogen uptake, as represented by a marginally increased specific surface area (from 750 to 820m^2^ g^−1^ for C‐HAT‐700 and 505 to 520m^2^ g^−1^ for C‐HAT‐550). The pore size distributions obtained from the quenched‐solid density functional theory (QSDFT) model^[22]^ show that all obtained samples contain a significant volume of micropores centered at around 1 nm and a small mesopore volume (Figure 3b). These results confirm that ball milling does not cause significant changes to the pore structure of the C_2_N‐type materials that is detectable by nitrogen physisorption measurements.

**Figure 4:**
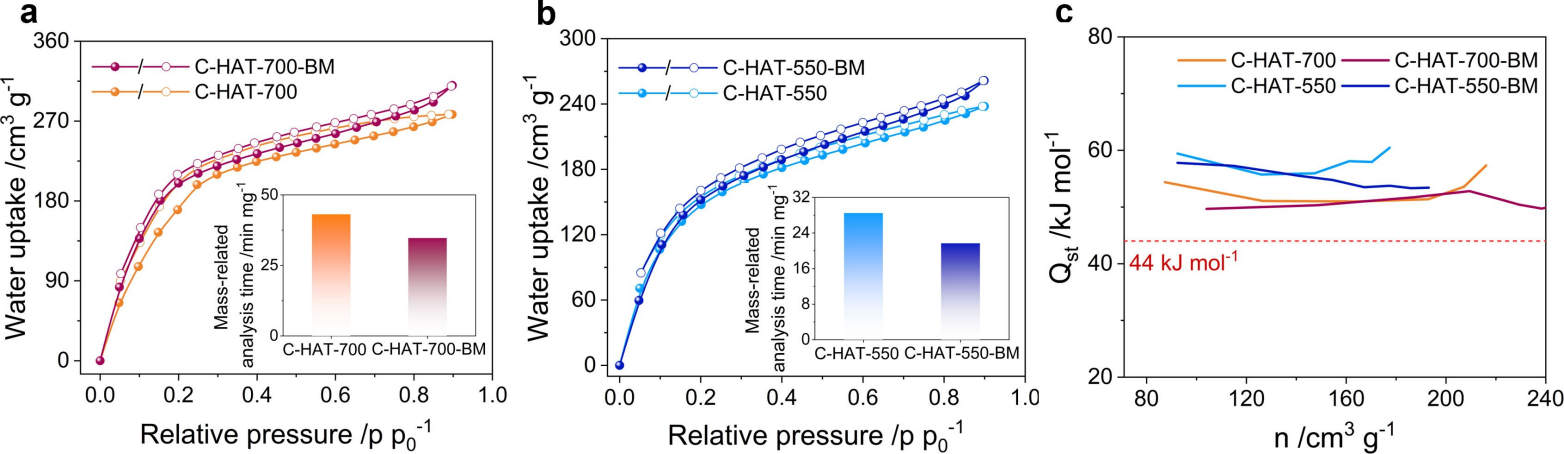
H_2_O vapor physisorption isotherms at 298 K for (a) C‐HAT‐700 and C‐HAT‐700‐BM; (b) C‐HAT‐550 and C‐HAT‐550‐BM. (Solid and empty circles represent adsorption and desorption data, respectively; Inserts show the mass‐related analysis time required for completing the adsorption/desorption isotherms. (c) The isosteric heats of water adsorption as a function of water uptake on C‐HAT‐700 and C‐HAT‐550 with and without ball milling. The dotted line refers to the heat evaporation of bulk water.

Furthermore, water vapor physisorption measurements at 298 K were carried out to evaluate the interaction of the samples with water (Figure 4a‐b). Type I isotherms with a steep capacity gain at low relative humidity are measured for both C_2_N‐type materials, suggesting the filling of narrow hydrophilic micropores. Such strong hydrophilic character and high water uptake is attributed to the abundant nitrogen functionalities[^11^, ^12b^] and the high micropore volume. It is worth noting that even though both isotherms have similar shapes, slightly higher total uptakes at high relative humidity (p p_0_^−1^>0.8) are observed for ball‐milled samples. This phenomenon is possibly caused by the breaking of carbon particles, thus creating more inter‐particular voids between the small fragments, allowing the adsorption of more water molecules close to the condensation pressure. Additionally, the sample obtained at 700 °C shows a slightly higher water uptake during adsorption at low relative pressure (p p_0_^−1^<0.2) after ball milling. In contrast, the desorption branches of C‐HAT‐700 and C‐HAT‐700‐BM have a precise overlap in this range. This difference has to be ascribed to the faster water adsorption after ball milling, that is, the reduced amount of time needed to reach the adsorption equilibrium, as the nitrogen functional sites as well as the micropore structure almost kept unchanged during ball milling according to the XPS and N_2_ physisorption analysis. Thus, to further evaluate the relative adsorption rate of different samples, the analysis time required for completing the whole physisorption measurement was investigated. After loading comparable weights of activated materials into the measurement cells, the time normalized by the mass of sample for completing the adsorption/desorption isotherms under the same protocol significantly decreased from 43.1 min mg^−1^ for C‐HAT‐700 to 34.7 min mg^−1^ for C‐HAT‐700‐BM and 28.5 min mg^−1^ for C‐HAT‐550 to 21.7 min mg^−1^ for C‐HAT‐550‐BM, in spite of nearly similar water uptakes. The shorter equilibration times needed for the ball‐milled samples can be associated with the smaller particle sizes after that lead to enhanced adsorption kinetics.

Volumetric water vapor physisorption at different temperatures (298 and 308 K) was performed to calculate the isosteric heats of adsorption (Q_st_) using the desorption curves of the isotherms (Figure S8). Both materials investigated in this work exhibit high Q_st_ values over the entire range of adsorbed water amount due to the strong interactions between C_2_N‐type materials and water (Figure 4c). Given that the enthalpy of evaporation of water corresponds to ∼44 kJ mol^−1^, it becomes evident that carbon‐water interactions in C_2_N are dominating over water‐water interactions. In comparison to C‐HAT‐700, C‐HAT‐550 exhibits higher Q_st_ values at a given water uptake. This results from the higher polarity due to the higher content of nitrogen functional groups. Overall, the volumetric water vapor physisorption measurements demonstrate that the respective samples prepared at the same synthesis temperature have nearly similar water adsorption properties under equilibrium conditions. There are already some hints to differences in the water adsorption kinetics also in these measurements, but they are, in some cases, so little obvious, that they even fall within the range of the experimental error of such measurements. A less time‐consuming method that allows clearer insights into these differences is therefore needed.

**Figure 5:**
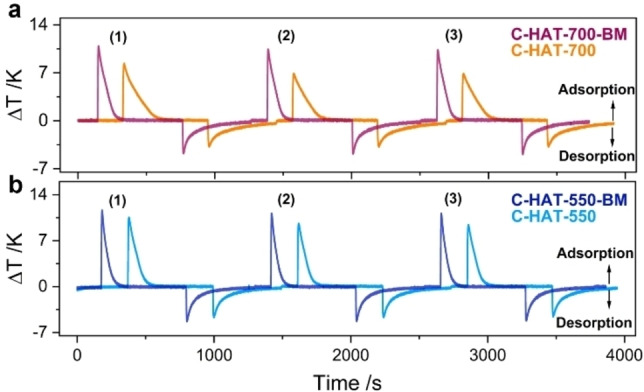
Thermal response measurement of H_2_O vapor adsorption/desorption cycling on (a) C‐HAT‐700 and C‐HAT‐700‐BM; (b) C‐HAT‐550 and C‐HAT‐550‐BM at 298 K (horizontally shifted for better visibility).

Thermal response analysis based on optical calorimetry is an attractive tool to provide direct insights into adsorption kinetics and uptakes within short times and in a relatively simple experimental setup.^[23]^ The InfraSORP technique tracks the time‐resolved temperature change of a sample taking place during the adsorption and desorption of a test adsorptive under dynamic flow conditions in real time with an infrared sensor (Figure 5).[^15a^, ^23a^] Here, wet nitrogen at 1 bar was used as the test gas and dry nitrogen at 1 bar as the flushing gas. The high affinity of all the samples to water becomes obvious by the significant heat release during adsorption. Due to the real‐time measurement with the optical calorimeter, the temperature signal can be directly used to draw conclusions on the transport properties of the materials for water vapor. For the two nitrogen‐rich carbon materials condensed at 700 and 550 °C, sharper adsorption peaks can be observed after ball milling, thus indicating the enhanced transport properties for the capture of water. While this difference caused by particle sizes is certainly expected and as such not surprising, we would like to point out here that the thermal response measurements are able to resolve and quantify these differences in a less time‐consuming way in comparison to the volumetric measurements.

C‐HAT‐1000 obtained at a higher condensation temperature of 1000 °C has been applied as additional reference material with lower nitrogen content.^[11]^ The weaker interaction between this rather graphitic carbon and water molecules leads to faster occupation of the adsorption sites, thus the enhancement of water adsorption kinetics by ball milling is not obvious anymore (Figure S9). This further confirms the assumption that the strong affinity to water as present in C‐HAT‐550 and C‐HAT‐700 leads to slower intraparticle diffusion rates. A modified thermal response function [Eq. (1)], including the migration of molecules from large voids to the microporous adsorption sites, can be used to calculate the adsorption rate constants, *k*_1_, of the microporous samples (details are described in the experimental section). The good quality for the initial signal of measured calorimetric data (R^2^>0.991) suggests the validity of the model for the selected samples (Figure S10). Independent of the condensation temperature, ball‐milled samples exhibited higher adsorption rate constants (*k*_1_=0.036 s^−1^ and 0.039 s^−1^ for C‐HAT‐700‐BM and C‐HAT‐550‐BM, respectively) compared to pristine C_2_N‐type materials (*k*_1_=0.026 s^−1^ and 0.032 s^−1^ for C‐HAT‐700 and C‐HAT‐550, respectively). It is thus found that the relative increase in the adsorption rate constant due to ball milling is slightly higher for the sample condensed at 700 °C.

Furthermore, previous investigations during repeated adsorption/desorption process using the InfraSORP revealed that this technique offers the opportunity to analyze the stability of a material towards repeated water adsorption and desorption.^[15b]^ Water desorption from the materials was initiated by flushing with a flow of 100 mL min^−1^ dry nitrogen for 500 s. As the flushing time was obviously not long enough to remove all adsorbed water molecules, the amount of adsorbed water decreased during the second adsorption cycle, as indicated by the lower thermal response signal intensity for all samples. C‐HAT‐700‐BM and C‐HAT‐550‐BM reached as much as 89 % and 94 % of their initial peak area, respectively. In contrast, only 77 % and 85 % of the initial adsorption capacity of untreated C‐HAT‐700 and C‐HAT‐550 can be recovered after the same desorption time. This observation confirms the slower transport of the water molecules in the non‐milled materials, leading to a lower degree of pore emptying. Furthermore, the faster adsorption/desorption process in the ball‐milled carbons is likely related to shorter diffusion paths in the pores as a consequence of the particle size reduction during ball milling, enabling an overall faster occupation of the adsorption sites by the adsorbate. Additionally, the amount of adsorbed water is proportional to the first intensity of temperature response signal and can be compared for C‐HAT‐700 and C‐HAT‐550 due to negligible variations in heat conductivity of the materials.^[16]^ The mass‐related integrated intensity of C‐HAT‐700 (119.8 K s mg^−1^) is higher than that of C‐HAT‐550 (98.4 K s mg^−1^, Figure S11), suggesting the higher water adsorption capacity for C‐HAT‐700. However, the nearly similar mass‐related integrated intensity before and after ball milling indicates no significant change for the water uptakes of the parent materials caused by the mechanical treatment. Both findings are in accordance with the volumetric water vapor isotherms. In summary, the thermal response measurements show the advantage of the ball‐milled carbon in terms of rapid water molecular transfer without compromising the adsorption capacity.

Finally, CO_2_ was also investigated as adsorbate to confirm the possibility to show the influence of smaller particle sizes on adsorption kinetics. Considering the strong interactions between CO_2_ and polar nitrogen functionalities, a different temperature signal becomes evident for nitrogen‐rich C‐HAT‐550 and C‐HAT‐700 samples after ball milling, while no notable difference can be seen for C‐HAT‐1000 after ball milling (Figure S12).

## Conclusion

The influence of the particle size on the kinetics of water adsorption on nitrogen‐rich C_2_N‐type materials with ultra‐high hydrophilicity was investigated. Ball milling of the particles present in the as‐made carbons leads to a decrease of the particle size, while maintaining the original architecture of the materials containing narrow micropores with nitrogen‐containing pore walls. Results of volumetric water adsorption as well as thermal response measurements show that the ball‐milled samples have the expected overall faster kinetics in the capture of water molecules as shown by the shorter equilibrium time required in the physisorption measurements and the faster water removal and sharper temperature responses in the InfraSORP measurement. The enhanced adsorption kinetics resulting from a shortening of the diffusion pathways at smaller particle size is further confirmed by the significantly higher adsorption rate constants of C‐HAT‐700‐BM/C‐HAT‐550‐BM (0.036 s^−1^/0.039 s^−1^) as compared to that of pristine C‐HAT‐700/C‐HAT‐550 (0.026 s^−1^/0.032 s^−1^). The similar integrated heat signals measured using the InfraSORP technique show that there is no loss of water adsorption capacity after ball milling treatment. On the one hand, this work can serve as a starting point for the investigation of the water adsorption kinetics of other materials with differences in their adsorption/desorption kinetics for water and other polar gases with strong adsorption. In contrast to the top‐down approach reported here, templating approaches seem to be especially promising for the synthesis of C_2_N‐like materials with pore hierarchy on the nanometer scale. On the other hand, this work proves and extends the possibilities provided by the InfraSORP technology for the analysis of water adsorption kinetics and reversibility on polar surfaces and could be expanded towards materials beyond nanoporous carbons.

## Experimental Section

**Materials Synthesis**. Typically, about 300 mg HAT‐CN (synthesized according to a previously described procedure)^[24]^ were condensed into the isothermal zone of a horizontal tubular furnace at 550, 700 or 1000 °C for 1 h under flowing N_2_ atmosphere. The temperature was firstly increased from room temperature to 80 °C with a heating ramp of 2 °C min^−1^, and then further increased to targeted temperature with 4 °C min^−1^. The condensed materials were named as C‐HAT‐X, where X stands for the maximum condensation temperature. After that, about 100 mg of C‐HAT‐X samples and three zirconia balls were placed in a zirconia jar of 50 mL size and then put in a Retsch PM 100 planetary for ball milling for 150 min at 500 rpm. The resulting materials were labelled as C‐HAT‐X‐BM.

**Characterization**. X‐ray diffraction (XRD) measurements were performed on Bruker D2 Advance X‐ray powder diffractometer (Germany) in a 2Θ range of 10–80° with Cu−Kα radiation (*λ*=0.154 nm). The samples were placed on a horizontal silicon single crystal holder. Scanning electron microscopy (SEM) measurements were taken with a Tescan LYRA XMU SEM at 5 kV acceleration voltage. The size distributions of the particle agglomerates were measured based on SEM micrographs by tracing the dimensions with a stylus and averaging the major and minor axis lengths. The static light scattering (SLS) measurements were performed on a Mastersizer 2000 analyzer with a Hydro 2000 S automatic dispersion unit and submicron instrumentation by Malvern instruments (United Kingdom). A light source with a wavelength of *λ*=633 nm was used. Purified water was selected as dispersant. Five runs were performed for each sample to get the average particle size distribution. X‐ray photoelectron spectroscopy (XPS) tests were carried out on a K‐Alpha spectrometer by ThermoFisher. The X‐ray source is an Al Kα anode (hν=1486.6 eV) and the powder samples were fixated on adhesive copper tape. Survey scans were measured using a step size of 1 eV in a range of 136.6–1360 eV and used to estimate the elemental composition. High resolution spectra were recorded with a step size of 0.5 eV as 5 averaged scans. The deconvolution of the XPS spectra on nitrogen‐rich carbons refers to the previous reported work.^[25]^ Spectra have not been referenced to C1s, the shifting of the binding energy was less than +0.3 eV. Charge compensation during measurement was realized by using a flood gun. Raman spectra were obtained using a Witec Raman Microscope spectrometer with an excitation wavelength of 633 nm at a laser power of 3.5 mW.

**Adsorption Measurements**. Prior to all measurements, the samples were degassed at 150 °C under vacuum for 12 h. Then, 22.3 mg of C‐HAT‐700, 21.2 mg of C‐HAT‐700‐BM, 24.7 mg of C‐HAT‐550 and 30.9 mg of C‐HAT‐550‐BM were packed into the measurement cell, respectively. Water vapor adsorption/desorption isotherms were measured volumetrically using a Autosorb IQ apparatus (Quantachrome Instruments). The analysis temperature was controlled by a circulating‐bath temperature controller with an accuracy of ±0.1 K. The isosteric heat of adsorption was calculated based on the Clausius‐Clapeyron equation using the option integrated in the ASiQwin 3.0 analysis software. Nitrogen physisorption was performed on a Quadrasorb SI apparatus (Quantachrome Instruments) at 77 K. The pore size distributions were calculated using quenched solid density functional theory (QSDFT) model provided by the ASiQwin 3.0 analysis software. The specific surface areas were calculated using the multi‐point Brunauer‐Emmett‐Teller (BET) model (p p_0_^−1^ range from 0.005 to 0.05).

**InfraSORP Adsorption/Desorption Cycling**. Thermal response measurements were performed using the InfraSORP instrument (Fraunhofer IWS, Dresden). All measurements were carried out at 1.0 bar and 298 K. Before each measurement, about 10 mg of the sample were placed into the sample holder and preheated at 150 °C under vacuum for 12 h. After cooling to room temperature, the sample cell was transferred into the instrument and purged with dry nitrogen flow until a stable temperature was reached. Then, a nitrogen flow saturated with water vapor (100 mL min^−1^) passed through the adsorbent for 500 s and the thermal response from the adsorption process was recorded. The wet gas has been generated by bubbling the nitrogen through a closed water reservoir at room temperature prior to supply to the InfraSORP device. After that, the cell was purged again with a dry nitrogen flow for another 500 s for desorption. The by‐pass time between the adsorption and desorption cycles has been set to 120 s. For the carbon dioxide measurements, a similar procedure was followed. Wet nitrogen has been replaced with CO_2_ as the test gas.

A fit of thermal response function [Eq. (1)] is proposed:^[26]^

(1) $$\Delta T\left(t\right)=\Delta T{}_{1}[\left(1-e{}^{-k1t}\right)-\left(1-e{}^{-k2t}\right)+\Delta T{}_{2}\left[\left(1-e{}^{-k3t}\right)-\left(1-e{}^{-k4t}\right)\right]]$$

Where Δ*T*(t) and t refer to the time‐resolved temperature signal and measurement time, respectively; Δ*T*_1_ (Δ*T*_2_) represents the maximum adiabatic temperature (of the migration process); *k*_1_ (*k*_3_) is the thermal rate constant (of the migration) and *k*_2_ (*k*_4_) is the thermal rate constant of heat transfer mechanism of convective cooling (for the migration process).

## Conflict of interest

The authors declare no conflict of interest.

1

## Supporting information

As a service to our authors and readers, this journal provides supporting information supplied by the authors. Such materials are peer reviewed and may be re‐organized for online delivery, but are not copy‐edited or typeset. Technical support issues arising from supporting information (other than missing files) should be addressed to the authors.

Supporting Information

## Data Availability

The data that support the findings of this study are available from the corresponding author upon reasonable request.
