# Supplementary material for: Application of Thermal Response Measurements to Investigate Enhanced Water Adsorption Kinetics in Ball‐Milled C2N‐Type Materials
Source: ChemistryOpen. 2022 Dec 13;11(12):e202200193. doi: 10.1002/open.202200193 (PMC9746058; doi:10.1002/open.202200193)
Supplement: Supplementary file 1 — Supporting Information [file OPEN-11-e202200193-s001.pdf]

ChemistryOpen

Supporting Information

Application of Thermal Response Measurements to Investigate Enhanced Water Adsorption Kinetics in Ball-Milled C_2N-Type Materials

Shengjun Du, Desirée Leistenschneider, Jing Xiao, Jan Dellith, Erik Troschke, and Martin Oschatz*

ChemistryOpen

Supporting Information

Application of Thermal Response Measurements to Investigate Enhanced Water Adsorption Kinetics in Ball-Milled C_2N-type Materials

Shengjun Du, Desirée Leistenschneider, Jing Xiao, Jan Dellith, Erik Troschke, and Martin Oschatz*

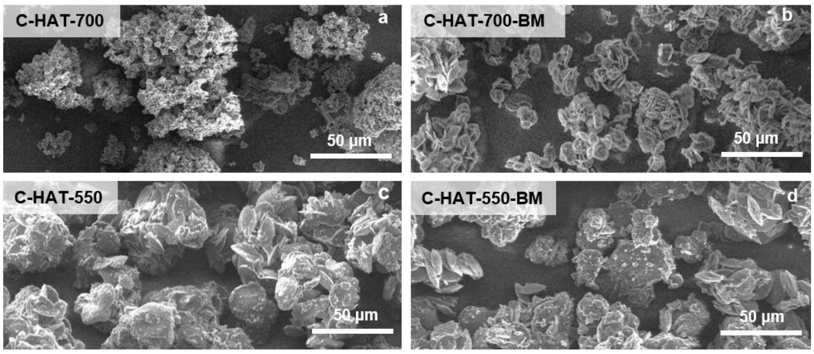

Figure S1. SEM images of C-HAT-700 and C-HAT-550 with and without ball milling treatment.

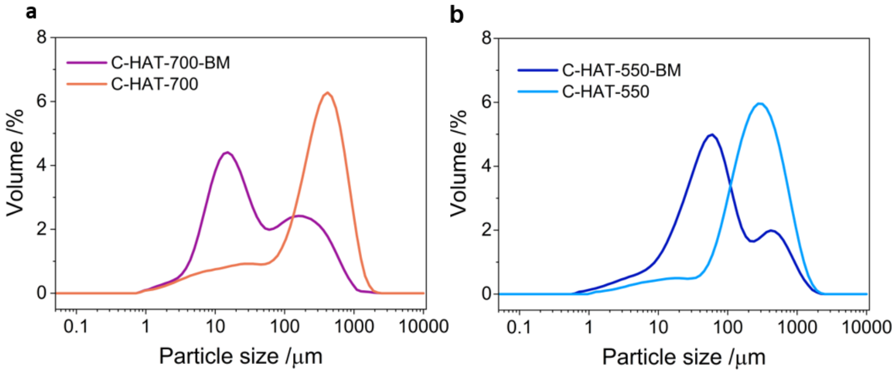

Figure S2. Particle size distributions of (a) C-HAT-700 and C-HAT-700-BM; (b) C-HAT-550 and C-HAT-550-BM based on the SLS technique.

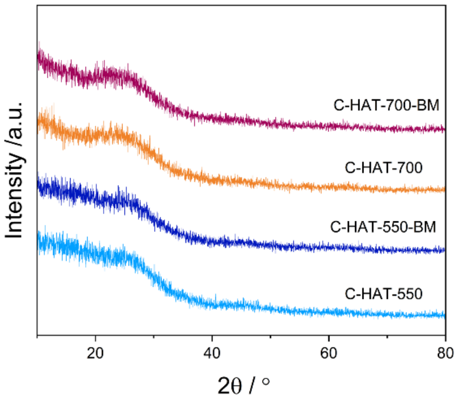

Figure S3. XRD patterns of C-HAT-700 and C-HAT-550 with and without ball milling treatment.

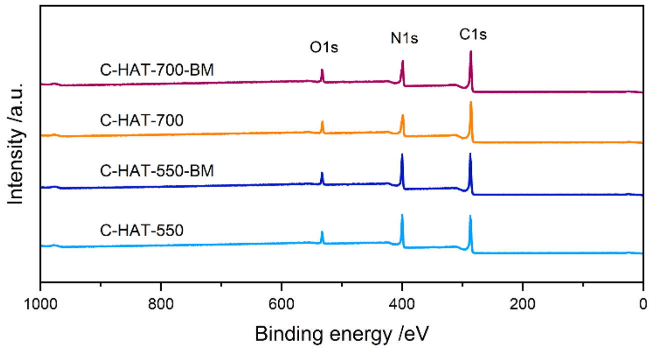

Figure S4. X-ray photoelectron spectroscopy survey spectra of C-HAT-700 and C-HAT-550 before and after ball milling.

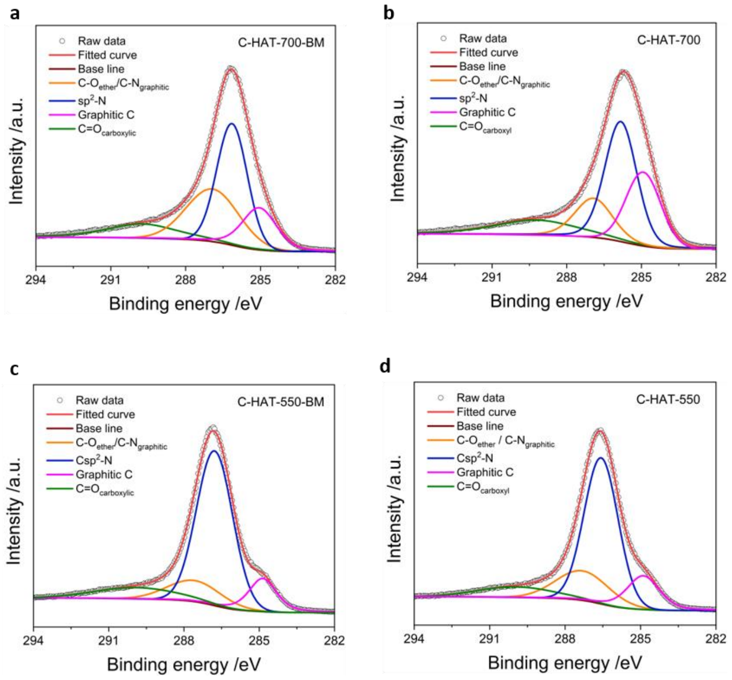

Figure S5. Fitted C1s XPS line scans of (a) C-HAT-700-BM; (b) C-HAT-700; (c) C-HAT-550-BM; (d) C-HAT-550.

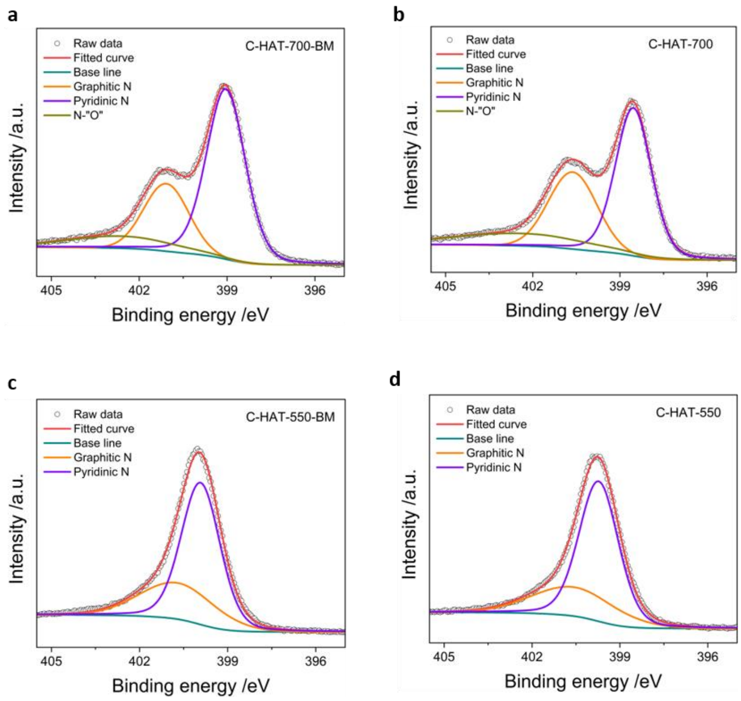

Figure S6. Fitted N1s XPS line scans of (a) C-HAT-700-BM; (b) C-HAT-700; (c) C-HAT-550-BM; (d) C-HAT-550.

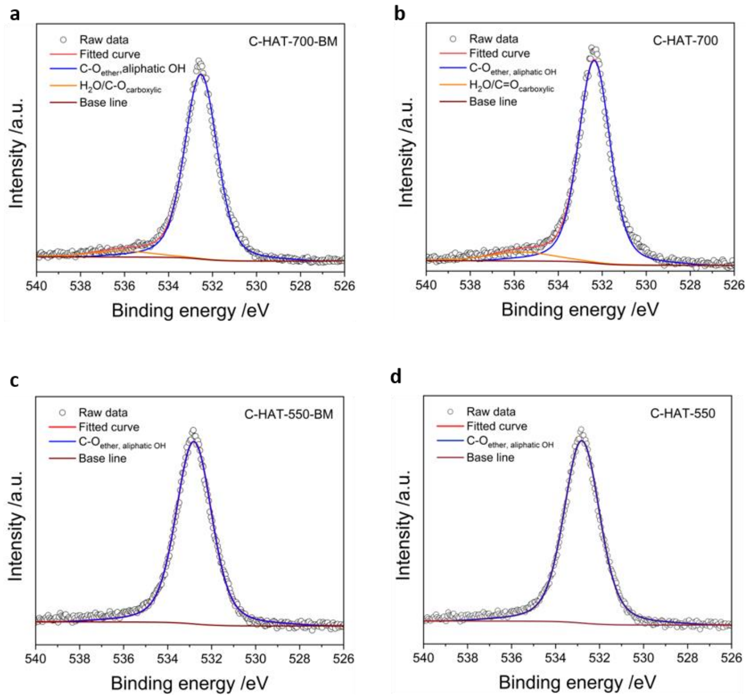

Figure S7. Fitted O1s XPS line scans of (a) C-HAT-700-BM; (b) C-HAT-700; (c) C-HAT-550-BM; (d) C-HAT-550.

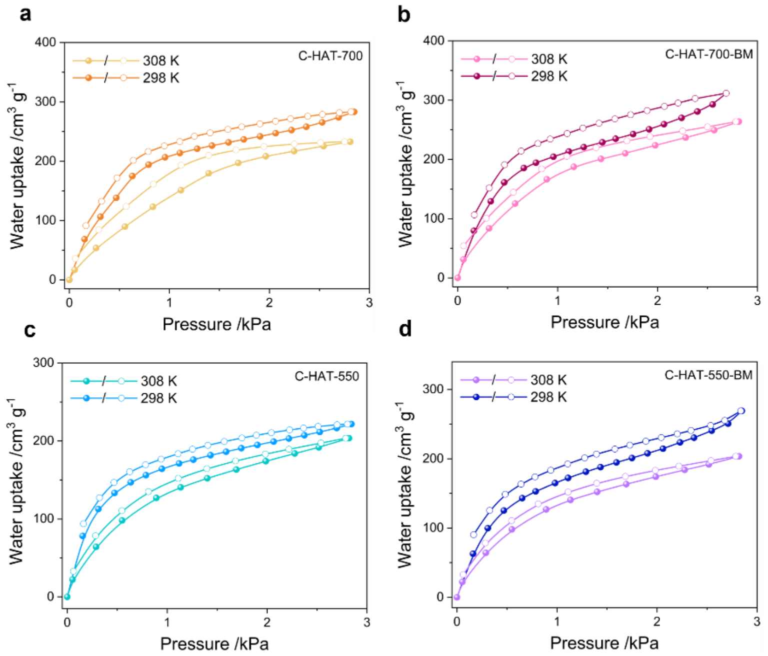

Figure S8. Water vapor physisorption isotherms at different temperatures (298 K and 308 K) of (a) C-HAT-700; (b) C-HAT-700-BM; (c) C-HAT-550; (d) C-HAT-550-BM.

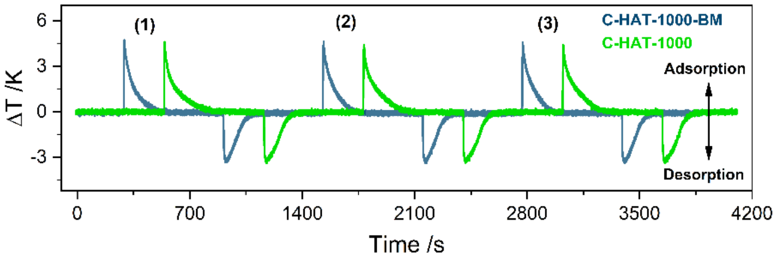

Figure S9. Thermal response measurement of H_2O vapor adsorption/desorption cycling on C-HAT-1000 and C-HAT-1000-BM at 298 K (horizontally shifted for better visibility).

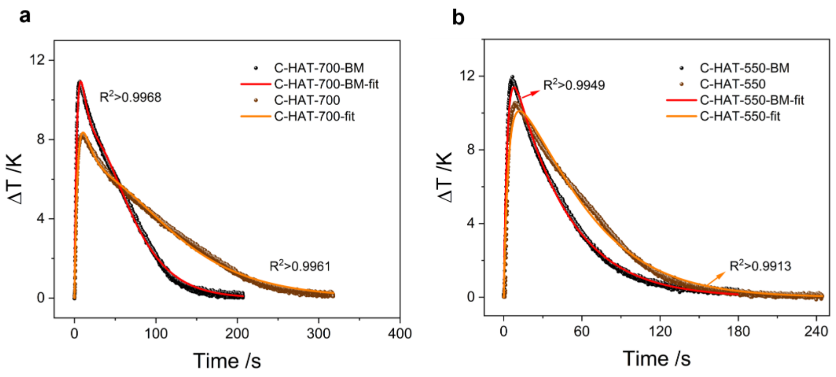

Figure S10. Fitting curves (straight lines) of the single peak thermal response function (Equation 1) to the measured data (dotted black lines) on (a) C-HAT-700-BM and C-HAT-700; (b) C-HAT-550-BM and C-HAT-550.

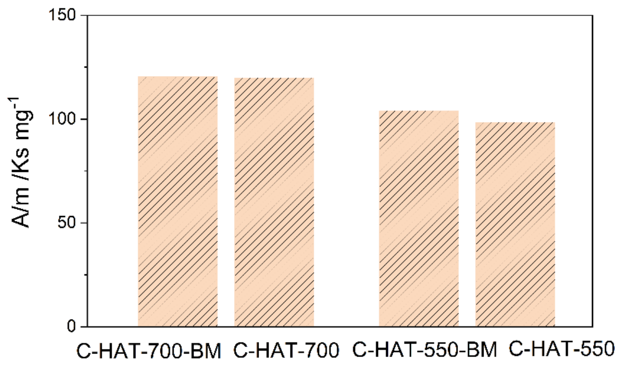

Figure S11. The mass-related integrated intensity of C-HAT-700 and C-HAT-550 with and without ball milling treatment in the thermal response measurement.

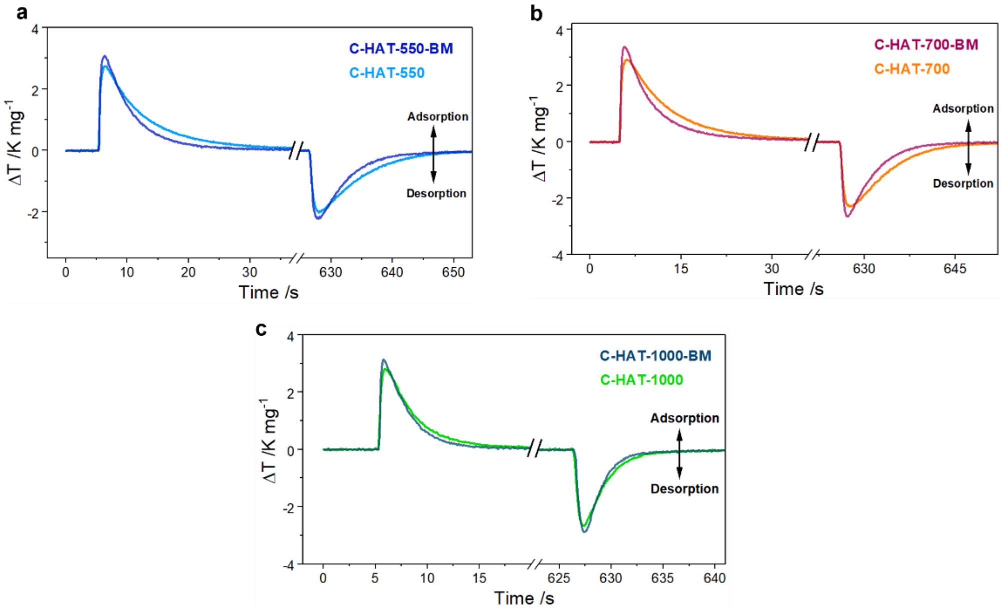

Figure S12. Normalized thermal response signal of CO_2 adsorption/desorption at 298 K on C-HAT-X before and after ball-milling based on InfraSORP technique.

Table S1. Elemental composition determined by XPS.

Materials	C /at%	N /at%	O /at%	C:N /at%	C:O /at%
C-HAT-700	66.0	26.9	7.0	2.5	9.4
C-HAT-700-BM	64.9	26.1	9.0	2.5	7.2
C-HAT-550	62.9	29.6	7.5	2.1	8.4
C-HAT-550-BM	62.0	30.5	7.5	2.0	8.3
